# Does Maternal Normal Range Thyroid Function Play a Role in Offspring Birth Weight? Evidence From a Mendelian Randomization Analysis

**DOI:** 10.3389/fendo.2020.601956

**Published:** 2020-11-12

**Authors:** Xinghao Zhang, Pengfei Wu, Yuyao Chen, Wan Zhang, Kun Xia, Huiyu Hu, Ping Zhou

**Affiliations:** ^1^ Department of Ultrasound, Third Xiangya Hospital, Central South University, Changsha, China; ^2^ Center for Medical Genetics, School of Life Sciences, Central South University, Changsha, China; ^3^ Department of Obstetrics and Gynecology, First People’s Hospital of Yueyang, Yueyang, China; ^4^ Department of Biology, College of Arts & Sciences, Boston University, MA, United States; ^5^ Center for Excellence in Brain Science and Intelligence Technology, Shanghai, China; ^6^ Department of Thyroid Surgery, Xiangya Hospital, Central South University, Changsha, China; ^7^ Department of Surgery, Massachusetts General Hospital, Harvard Medical School, Boston, MA, United States

**Keywords:** thyroid function, birth weight, Mendelian randomization, causality, genetic epidemiology

## Abstract

**Background:**

The association between normal range thyroid function and offspring birth weight has been postulated, but evidence from observational studies is prone to be confounded. We conducted a two-sample Mendelian randomization (MR) study to explore the causal effects of maternal thyroid stimulating hormone (TSH) and free thyroxine (FT4) on birth weight.

**Methods:**

We utilized public shared summary-level statistics from European-ancestry genome wide association studies. We obtained 40 and 21 single nucleotide polymorphisms as instrumental variables, which were associated with TSH and FT4 levels at genome-wide significance (*P* < 5 × 10^−8^). Partitioned maternal effects on birth weight were retrieved from datasets contributed by the Early Growth Genetics Consortium. Inverse-variance weighted method was employed in the primary MR analysis and multiple sensitivity analyses were implemented.

**Results:**

Genetically determined normal range thyroid function was not causally associated with offspring birth weight. Each one standard deviation (SD) increase in maternal TSH was associated with 0.002 SD higher of birth weight (95% confidence interval [CI], −0.021 to 0.025; *P* = 0.87). Similarly, change in birth weight was −0.001 SD (95% CI, −0.031 to 0.029; *P* = 0.94) per one SD higher in maternal FT4. Consistent results were yielded *via* additional MR methods. Sensitivity analyses demonstrated no presence of horizontal pleiotropy or heterogeneity.

**Conclusion:**

This MR study did not identify a causality between normal range thyroid function and offspring birth weight in the Europeans.

## Introduction

Thyroid dysfunction features altered thyroid stimulating hormone (TSH) and free thyroxine (FT4) concentrations (1) and is involved in pathophysiological conditions of multiple systems (2–7). In addition to overt thyroid disease, mild thyroid function abnormality, such as subclinical hyperthyroidism and hypothyroidism with normal range FT4 levels, and isolated hypothyroxinemia in the setting of normal TSH levels, has broad implications as well (8, 9). Birth weight is an essential predictor of intrauterine exposures and infant health. Recent studies suggested that normal thyrotropin within the upper reference range or subclinical hypothyroidism may exert positive (10, 11), negative (12, 13), or null (14, 15) effects on offspring birth weight. Apart from the inconsistency, evidence from previous case-control or prospective cohort studies was prone to various confounders. Thus, whether maternal normal range TSH or FT4 levels are associated with newborn birth weight, and whether large or small for gestational age, remains inconclusive.

Mendelian randomization (MR) has been advancing as a powerful genetic-epidemiological tool to make causal inference and yield robust estimate (16). MR studies employs single nucleotide polymorphisms (SNPs) which are identified from genome-wide association studies (GWAS) as instrumental variables for biological traits of interest. By courtesy of Mendel’s laws, independent assortment of alleles during gamete formation renders a more natural and ideal randomization than in randomized controlled trials. MR studies are capable of giving evidence of high-level strength and adequate power, especially when sufficiently large sample size of mother–offspring duos is hardly feasible (17, 18). Here we performed a two-sample MR study to investigate the association between maternal normal range TSH and FT4 with offspring birth weight.

## Materials and Methods

### Overall Design

**Figure 1:**
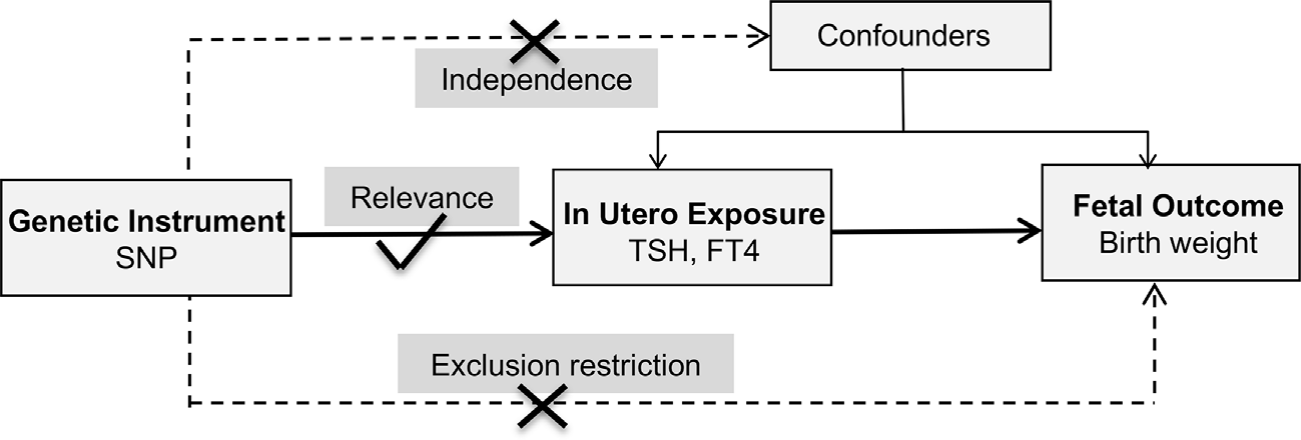
Schematics for the Mendelian randomization study. FT4, free thyroxine; SNP, single nucleotide polymorphism; TSH, thyroid stimulating hormone.

This study adopted a two-sample MR design and publicly shared datasets. No additional ethic approval or informed consent *was required.* The framework underlying this MR study was shown in **Figure 1**. Causal effects of maternal TSH and FT4 on the offspring birth weight were explored separately. Three fundamental assumptions were examined first. The relevance assumption was satisfied since these variants were associated with thyroid function at genome-wide significance level (*P* < 5 × 10^−8^). Then, the independence assumption was verified considering the intrinsic strength of MR design. Confounding factors which could influence gamete formation and randomized segregation and independent assortment of alleles scarcely existed. Furthermore, the exclusion–restriction assumption was examined through multiple sensitivity analyses. Two-sample MR also required that instruments proxied for thyroid function were not in linkage disequilibrium to minimize bias on the overall causality estimate. Finally, a set of instrumental variables that were uncorrelated with each other were established.

### Data Source

Summary statistics for TSH and FT4 were extracted from the European-ancestry GWAS meta-analysis on thyroid function within the normal range by Teumer et al. (19). Totally, 72167 European participants without medical record of thyroid medication or surgery, were incorporated into this original study. At genome-wide significance level (*P* < 5 × 10^−8^), 61 lead SNPs at 42 loci were identified for circulating TSH levels (**Supplemental Table 1**), whereas 31 lead SNPs at 21 loci associated with circulating FT4 levels (**Supplemental Table 2**). In the original GWAS, index SNPs at each locus were independent without linkage disequilibrium (r^2^ *< 0.8)*. Since a stringent threshold was required in MR analysis using multiple instrumental variables, we further performed clumping (threshold set at r^2^ < 0.01, within 1Mb, the European panel, 1000 Genomes Project Phase 3) using LDlink, a web-based application (20), and retained the one with the lowest P-value in each locus. Finally, we obtained 40 and 21 SNPs as genetic proxies for serum concentrations of TSH (**Supplemental Table 3**) and FT4 (**Supplemental Table 4**), respectively. Effect size (Beta) represented change in the standard deviation (SD) of TSH (~0.8 mIU/L) and FT4 levels (~0.2 ng/dl) per additional effect allele.

For selected instrumental SNPs, we retrieved European-ancestry maternal-specific effect estimate on offspring’s birth weight (http://egg-consortium.org/birth-weight-2019.html). Warrington et al. (18) generated and released four summary-level datasets, own birth weight, offspring birth weight, adjusted fetal effect, and adjusted maternal effect. Here, the last one, partitioned mother’s genetic effect on offspring’s birth weight, which has accounted for the correlation between fetal and maternal genotypes, was used in the MR setting to explore causal effects of intrauterine exposures. Specifically, Warrington et al. (18) conducted a meta-analysis in European individuals mainly from the UK Biobank the Early Growth Genetics Consortium, including *mother-offspring duos* with both their own and offspring’s birth weight reported, and participants with own birth weight (*n* = 264,498) or with offspring’s birth weight (*n* = 179,360). Derived *from individual-level data of mother-offspring duos*, the structural equation model (17, 18) was established to partition genetic contributions into direct fetal and indirect maternal effects, which adjusted for genetic interactions due to maternal-fetal transmitted alleles. Warrington et al. (18) has generalized and validated the model, shared summary-level statistics of the maternal effect on birth weight, and made preliminary application to investigate causal effects of maternal glycemic traits on birth weight. Therefore, estimates for the maternal SNP-effect on birth weight, were used and regressed against SNP-effect on thyroid function in the following MR analysis. For all instrumental variants but one rs11039355 (**Supplemental Table 4**), corresponding SNPs were directly available in the birth weight dataset and proxied SNPs were not utilized. Birth weight has been normalized transformed, and Beta coefficients represented SD change (~454 g) per additional effect allele.

### Statistical Analysis

The MR analysis was conducted in R software, version 3.6.1 (R Foundation for Statistical Computing, Vienna, Austria.) using *TwoSampleMR* package (21). The fixed-effect inverse variance weighted (IVW) method was adopted as the primary MR approach to compute an overall estimate from multiple instrumental SNPs (22). Given individual SNP*_k_*, its genetic effect on the exposure ${\hat{\beta }}_{X_{k}}$ with the standard error $\sigma_{X_{k}}$, and the outcome-effect ${\hat{\beta }}_{Y_{k}}$ and $\sigma_{Y_{k}}$, the causal effect can be estimated by the Wald ratio ${\hat{\beta }}_{Y_{k}}/{\hat{\beta }}_{X_{k}}$ with the standard error $\sigma_{Y_{k}}/{\hat{\beta }}_{X_{k}}$. Then a meta-analysis yielded an overall causal estimator ${\hat{\beta }}_{IVW}$ with standard error ${\hat{\sigma }}_{IVW}$ as shown below.

$${\hat{\beta }}_{IVW}=\frac{\Sigma_{k}X_{k}Y_{k}\sigma_{Y_{k}}^{-2}}{\Sigma_{k}X_{k}^{2}\sigma_{Y_{k}}^{-2}}$$

$${\hat{\sigma }}_{IVW}=\sqrt{1/\Sigma_{k}X_{k}^{2}\sigma_{Y_{k}}^{-2}}$$

Two complementary MR approaches were implemented, weighted median estimator (23) and MR-Egger regression (24). Weighted median estimator gave the pooled effect size more accurately when more than half of instrumental variables are valid. MR-Egger method was based on the assumption that pleiotropic effects are independent of one another. MR-Egger regression intercept provided an assessment of horizontal pleiotropy, whereas the slope gave an estimate adjusted for unbalanced pleiotropy across all variants. Additional sensitivity analyses were performed using Cochran’s *Q* tests, leave-one-out plots and funnel plots. Power calculations were completed with *mRnd*, a web tool (25). Statistical significance was set at the 2-sided *P* value of 0.025 in accordance with Bonferroni correction.

## Results

### Effect of Maternal TSH on Birth Weight

**Figure 2:**
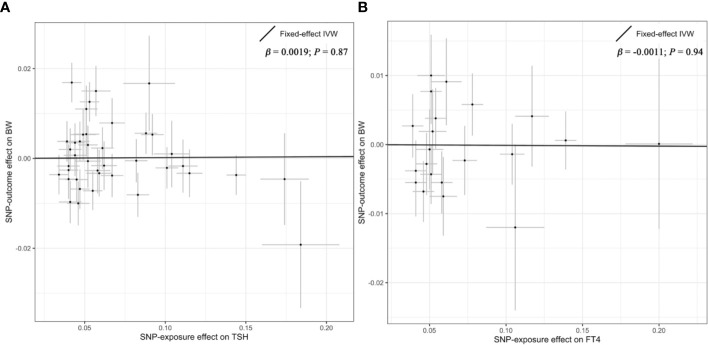
Primary results of the Mendelian randomization analysis. Individual SNP-effect on birth weight (point and vertical line) against its effect on TSH or FT4 (point and horizontal line) was delineated in the background. The causal estimate given by the IVW method (fitted line) for the effect of maternal TSH on birth weight was shown in **(A)**, while the overall effect for the casual association of maternal FT4 with birth weight was presented in **(B)**. FT4, free thyroxine; IVW, Inverse variance weighted; SNP, single nucleotide polymorphism; TSH, thyroid stimulating hormone.

**Figure 3:**
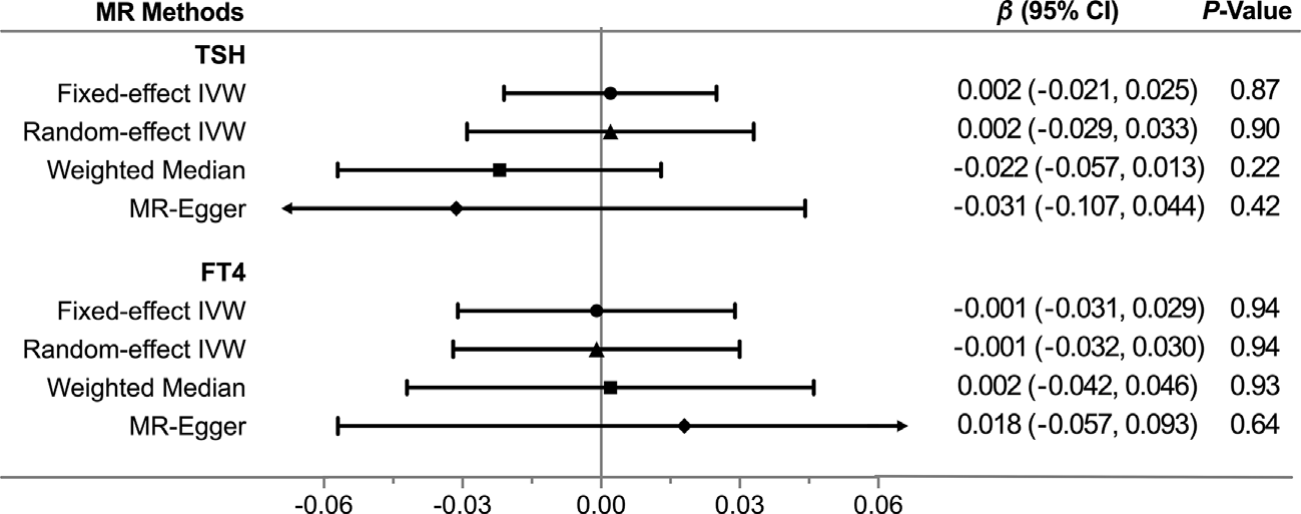
Comparisons of Mendelian randomization results by different methods. CI, Confidence interval; FT4, free thyroxine; IVW, inverse variance weighted; MR, Mendelian randomization; TSH, thyroid stimulating hormone.

**Figure 4:**
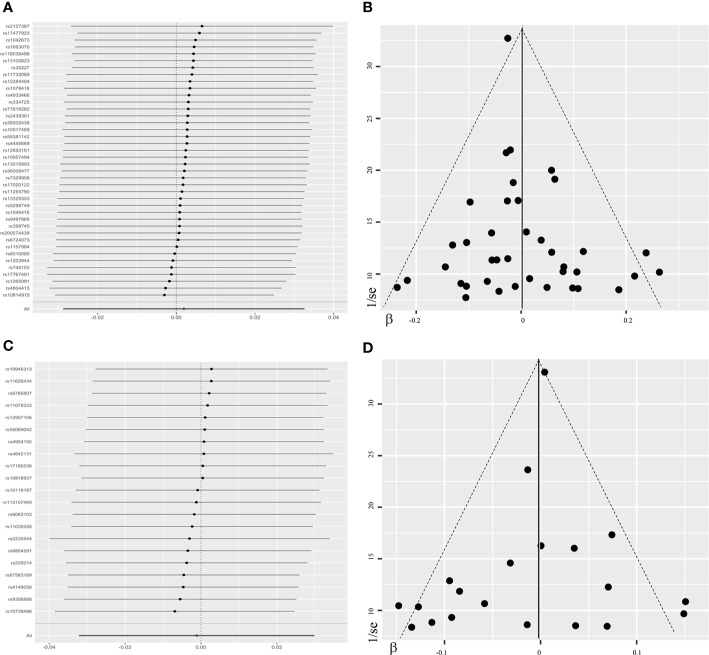
Leave-one-out plots and funnel plots in the sensitivity analyses. The leave-one-out plot **(A)** visualized how the causal estimates (point with horizontal line) for the effect of maternal TSH on birth weight were influenced by the removal of single variant. The funnel plot **(B)** illustrated the overall symmetry of causal estimates across all instrumental variables. Each point denoted corresponding inverse standard error against the individual causal estimate. The leave-one-out plot **(C)** and funnel plot **(D)** in the sensitivity analysis of FT4-birth weight relationship were presented likewise. FT4, free thyroxine; TSH, thyroid stimulating hormone.

Overall, there was no causal relationship between genetically predicted TSH concentrations and offspring birth weight. Primary MR results (**Figure 2A**) indicated that one SD increase in TSH levels was associated with 0.002 SD higher of birth weight by the fixed-effect IVW method (95% confidence interval [CI], −0.021 to 0.025; *P* = 0.87). Effect size estimates (SD change in birth weight per one SD higher in TSH) by the random-effect IVW, weighted median and MR-Egger method were 0.002 (*P* = 0.90), −0.022 (*P* = 0.22) and −0.031 (*P* = 0.42), respectively, which were roughly consistent (**Figure 3**). No horizontal pleiotropy was identified (**Table 1**) by MR-Egger test (*P* = 0.35) and there was no significant heterogeneity detected through Cochran’s *Q* test (*P* = 0.12). Elimination of single instrumental SNP would not lead to distortion of the overall MR estimate (**Figure 4A**), whereas overall symmetry of the funnel plot (**Figure 4B**) further demonstrated negligible heterogeneity and validated the robustness of the causal estimate given by the fixed-effect IVW method. *F*-statistic for individual variant ranged 64 to 496; hence, none was weak instrument (*F* < 10). Proportion of variance in TSH explained by 40 instrumental SNPs, approximated 9.4%. Assuming a power of 80% to be adequate (α = 0.05), we were not capable of detecting such a weak effect as ${\hat{\beta }}_{IVW}$ within ± 0.018.

**Table 1:** Primary statistics in Mendelian randomization sensitivity analyses.

Exposures	MR-Egger regression			Heterogeneity test^*^		
	Intercept	Standard Error	*P*-value	*Q-*statistic	*Q_df*	*P*-value
Thyroid stimulating hormone	2.48 × 10^−3^	2.61 × 10^−3^	0.35	71.36	39	0.12
Free thyroxine	−1.53 × 10^−3^	2.76 × 10^−3^	0.59	21.16	20	0.39

*Heterogeneity test was performed with the fixed-effect inverse-variance weighted approach.

MR, Mendelian randomization.

### Effect of Maternal FT4 on Birth Weight

Likewise, genetically determined maternal FT4 was not causally associated with the offspring birth weight. By the main MR method (**Figure 2B**), change in birth weight was −0.001 SD (95% CI, −0.031 to 0.029; *P* = 0.94) per one SD higher in maternal FT4. Causal estimates by three additional approaches (**Figure 3**) did not reach nominal significance, either. There was no evidence for the existence of horizontal pleiotropy or heterogeneity by the MR sensitivity analyses (**Table 1**). Examination of the leave-one-out plot and funnel plot (**Figures 4C, D**) suggested that the MR results were not driven by certain SNP alone and overall causal estimates were consistent and accurate on the whole. None of instrumental variants for FT4 was weak instrument (*F*-statistic, 77 to 609). Given the proportion of variance explained of 4.8% and the power set at 80%, the minimally-detectable effect size was ± 0.025.

## Discussion

Birth weight has long been postulated to affect susceptibility to diseases in later life, known as the fetal origin of adult diseases hypothesis (26–28). Identifying influences of maternal exposures on birth weight should be meaningful (18). Previous observational studies hinted at the role for mild abnormalities of thyroid function in birth weight, nonetheless, with heterogeneous results. Meanwhile, triangulating evidence across multiple study designs should be worthwhile when assessing the influence of maternal TSH and FT4 on birth weight. Therefore, we conducted the first MR study to provide supplementary evidence and effect estimates for the associations of normal range thyroid function with offspring birth weight. We failed to identify a statistically significant causality between genetically predicted TSH and FT4 concentrations and birth weight in the European population.

One recent cohort (12) incorporated 1521 European women with thyroid function tests in the first trimester, and manifested that women with normal range TSH levels at upper limit were related to lower neonatal birth weight, with an adjusted odds ratio in reference to the normal group of 21.38 (95%CI, 1.29 to 353.39; *P* = 0.032). In a real-word data setting or traditional observational design, it was virtually impossible to allow for and adjust for all cofounding conditions such as gestational diabetes. Previous effect estimates for the maternal-fetal relationship still warranted replications in large well-designed cohorts. Another recent meta-analysis (29) aggregated individual-level data of 48,145 mother–child pairs from 20 observational cohorts and detected an inverse association between maternal TSH and FT4 within the normal range and birth weight. Specifically, each 1 SD higher maternal TSH and FT4 was associated with 6 g lower (95%CI, −10 to −2; *P* = 0.003) and 21 g lower (95%CI, −25 to −17; *P* < 0.0001) birth weight, respectively. Notably, in addition to the European population, more than 20% participants were of Asian ancestry (China, Japan and Pakistan). Although Newcastle–Ottawa scale and *I^2^* statistic suggested low to moderate bias and heterogeneity in the meta-analysis, without stratified analysis we could not rule out the possibility of an overwhelmingly significant effect in the Asians alone, which overweighed the null effect in the Europeans. Besides, currently available GWAS statistics on normal range TSH and FT4 were in the general rather than female-specific population. Population and gender difference might be another explanation for the null finding in our study.

To enlarge the sample size in the MR setting, we utilized instrumental SNPs associated with lifelong circulating TSH or FT4 to surrogate gestational thyroid function, instead of directly trimester-specific measurement as in traditional cohorts. Reference intervals of thyroid function altered along with the gestation trimester (30), and individual TSH and FT4 levels during pregnancy could manifest minimum differences from pre-gestational ones. It raised concern that genetically predicted thyroid function here was not the most appropriate proxies yet. Nevertheless, the two-sample MR framework (18) still provided an ideal supplement for traditional observational and original MR studies which were built on restricted sample size of mother–offspring duos. Further GWAS—dedicated to reveal genetic contributions to maternal serum and placental TSH and FT4 levels during pregnancy, would pave the way for elucidating effects of uterine thyroid function on fetal growth.

Several limitations existed in this study. First, summary-level data-based MR could not ascertain non-linear associations between maternal thyroid function and birth weight. Thus, we could not rule out that normal range TSH or FT4 exerted its influence within extremely upper or lower limit. Second, common variants identified by current GWAS still explained a relatively small proportion of variance. Hence, this study had restricted power to identify weak associations. Thirdly, the current MR analysis was restricted in the European-ancestry datasets and great caution should be exercised when generalizing the conclusion. Lastly, we couldn’t rule out the possibility that genetic variants underlying thyroid function during pregnancy was different from the current instrumental-SNP set and to which extent the difference might be, and we failed to take into account sex stratification and age difference within two datasets, which might affect the MR estimates.

To conclude, this MR study did not support the casual effects of maternal normal range TSH and FT4 on offspring birth weight in the European population. Triangulation of evidence across cohort studies, meta-analyses and MR studies was deemed necessary as well.

## Data Availability Statement

The original contributions presented in the study are included in the article/**Supplementary Material**. Further inquiries can be directed to the corresponding author.

## Author Contributions

XZ, PW, HH, and PZ conceptualized the study. XZ, PW, YC, and WZ took part in the data curation, methodology, software, and formal analysis. XZ, PW, PZ, KX, and HH were in charge of the validation and visualization. All authors contributed to the article and approved the submitted version.

## Conflict of Interest

The authors declare that the research was conducted in the absence of any commercial or financial relationships that could be construed as a potential conflict of interest.
