# Supplementary material for: Does Maternal Normal Range Thyroid Function Play a Role in Offspring Birth Weight? Evidence From a Mendelian Randomization Analysis
Source: Front Endocrinol (Lausanne). 2020 Nov 12;11:601956. doi: 10.3389/fendo.2020.601956 (PMC7689005; doi:10.3389/fendo.2020.601956)
Supplement: Supplementary file 1 [file DataSheet_1.pdf]

Supplemental Material

Table S1 Genome-wide significant SNPs for thyroid stimulating hormone and SNPs selected for instrumental variables in Mendelian randomization analysis

Table S2 Genome-wide significant SNPs for free thyroxine and SNPs selected for instrumental variables in Mendelian randomization analysis

Table S3 Association of instrumental SNPs with thyroid stimulating hormone and birth weight

Table S4 Association of instrumental SNPs with free thyroxine and birth weight

Table S1 Genome-wide significant SNPs for thyroid stimulating hormone and SNPs selected for instrumental variables in Mendelian randomization analysis

No.	SNP	Chr	Position (GRCh37)	Nearest Gene	*P*-value	Exclusion*
1	rs12089835	1	19771438	CAPZB	1.30E-28	Clumping
2	rs10917469	1	19843576	CAPZB	4.00E-39	
3	rs74804879	1	19862320	CAPZB	1.20E-14	Clumping
4	rs334725	1	61610049	NFIA	2.40E-32	
5	rs17020122	1	108357391	VAV3	5.30E-20	
6	rs16856540	2	217580413	IGFBP5	7.80E-11	Clumping
7	rs13015993	2	217625523	IGFBP5	4.50E-32	
8	rs6724073	2	218236786	DIRC3	1.30E-10	
9	rs1663070	3	12239852	SYN2	3.50E-11	
10	rs28502438	3	149220109	TM4SF4	3.70E-08	
11	rs13100823	3	185514088	IGF2BP2	6.80E-10	
12	rs59381142	3	193916181	HES1	1.70E-14	
13	rs6535624	4	149587905	NR3C2	1.60E-11	Clumping
14	rs11732089	4	149665602	NR3C2	1.70E-51	
15	rs62362610	5	76439961	PDE8B	7.70E-10	Clumping
16	rs1119208	5	76488613	PDE8B	6.60E-13	Clumping
17	rs139424329	5	76495539	PDE8B	5.10E-10	Clumping
18	rs2127387	5	76532571	PDE8B	1.10E-117	
19	rs7702192	5	76554807	PDE8B	2.60E-30	Clumping
20	rs113974964	5	76652403	PDE8B	2.10E-17	Clumping
21	rs139149784	5	76660193	PDE8B	5.00E-08	Clumping
22	rs182873197	5	76773148	PDE8B	1.70E-08	Clumping
23	rs1265091	6	31108129	PSORS1C1	3.20E-11	
24	rs744103	6	43805362	VEGFA	6.70E-41	
25	rs9381266	6	43905037	VEGFA	1.80E-25	Clumping
26	rs9497965	6	148521292	SASH1	9.80E-13	
27	rs73022105	6	165973757	PDE10A	1.20E-11	Clumping
28	rs1079418	6	166047034	PDE10A	8.20E-53	
29	rs56009477	8	23356964	SLC25A37	3.70E-10	
30	rs2439301	8	32433013	NRG1	8.20E-15	
31	rs10957494	8	70365025	SULF1	1.10E-09	
32	rs118039499	8	133771635	TG	2.00E-14	
33	rs2739067	8	133951991	TG	2.40E-11	Clumping

34	rs10814915	9	4290544	GLIS3	5.10E-12	
35	rs9298749	9	16214340	C9orf92	8.80E-10	
36	rs8176645	9	136149098	ABO	3.90E-16	NA
37	rs11255790	10	8682180	GATA3	6.80E-10	
38	rs4933466	10	89849519	PTEN	5.10E-10	
39	rs200574439	10	101283330	NKX2-3	3.70E-13	
40	rs12284404	11	45228686	PRDM11	2.50E-22	
41	rs4445669	11	115045237	CADM1	5.80E-11	
42	rs7329958	13	24782080	SPATA13	1.10E-11	
43	rs398745	14	36536181	MBIP	4.00E-17	
44	rs2254613	14	36713154	MBIP	3.40E-08	Clumping
45	rs11159482	14	81490842	TSHR	6.30E-11	Clumping
46	rs59334515	14	81594143	TSHR	1.10E-13	Clumping
47	rs12893151	14	81619945	TSHR	1.00E-15	
48	rs8015085	14	93585331	ITPK1	2.40E-18	
49	rs17477923	15	49711185	FAM227B	2.60E-33	
50	rs11639111	15	49749735	FAM227B	3.60E-13	Clumping
51	rs13329353	15	89113877	DET1	5.20E-21	
52	rs1045476	16	4015313	ADCY9	2.40E-09	
53	rs30227	16	14405428	MIR365A	7.60E-14	
54	rs17767491	16	79745487	MAF	3.40E-42	
55	rs77819282	17	44762589	NSF	1.10E-09	
56	rs1157994	17	59338574	BCAS3	5.30E-09	
57	rs1042673	17	70121339	SOX9	3.60E-19	
58	rs963384	17	70369758	SOX9	2.80E-08	
59	rs4804413	19	7222655	INSR	8.60E-18	
60	rs1203944	20	22596879	FOXA2	2.40E-12	
61	rs12390237	X	3612081	PRKX	1.70E-11	NA

Note: ***** To retain an independent set of SNPs for Mendelian randomization analysis, clumping threshold was set at linkage disequilibrium $r^2 < 0.01$, within 1Mb, the European panel, 1000 Genomes Project Phase 3.

§Two variants were removed since, rs8176645 at the ABO locus has pleiotropic effects and rs12390237 at X chromosome was not present in the birth weight dataset.

Abbreviations: Chr, Chromosome; SNP, single nucleotide polymorphism.

Table S2 Genome-wide significant SNPs for free thyroxine and SNPs selected for instrumental variables in Mendelian randomization analysis

No.	SNP	Chr	Position (GRCh37)	Nearest Gene	*P*-value	Exclusion*
1	rs145019385	1	54252139	DIO1	1.10E-08	Clumping
2	rs12033572	1	54369674	DIO1	1.40E-11	Clumping
3	rs2235544	1	54375570	DIO1	4.20E-101	
4	rs954878	1	54578401	DIO1	4.80E-19	Clumping
5	rs4954192	2	135632981	ACMSD	8.40E-09	
6	rs6785807	3	181718601	SOX2-OT	2.50E-10	
7	rs6854291	4	170992760	AADAT	1.30E-24	
8	rs10946313	6	19381386	ID4	2.30E-11	
9	rs9356988	6	25777481	SLC17A4	3.60E-12	
10	rs137964359	6	26001742	SLC17A4	2.10E-10	Clumping
11	rs17185536	6	100620931	LOC728012	1.90E-19	
12	rs67583169	8	61212179	CA8	1.00E-10	
13	rs10119187	9	4223660	GLIS3	4.10E-09	
14	rs10739496	9	100552559	FOXE1	4.20E-30	
15	rs10984606	9	100739117	FOXE1	1.20E-09	Clumping
16	rs10818937	9	127015440	NEK6	1.30E-11	
17	rs4842131	9	139092679	LHX3	7.70E-44	
18	rs55679545	9	139122363	LHX3	8.40E-09	Clumping
19	rs11039355	11	47737501	FNBP4	3.50E-08	
20	rs4149056	12	21331549	SLCO1B1	1.30E-08	
21	rs150816132	14	80464293	DIO2	3.50E-08	Clumping
22	rs978055	14	80534869	DIO2	1.10E-08	Clumping
23	rs225014	14	80669580	DIO2	1.80E-15	

24	rs12323871	14	101852075	DIO3OS	1.40E-08	Clumping
25	rs11626434	14	101998443	DIO3OS	4.10E-17	
26	rs12907106	15	63873658	USP3	3.70E-08	
27	rs8063103	16	12703395	SNX29	1.60E-08	
28	rs11078333	17	16049626	NCOR1	9.90E-13	
29	rs1080094	18	29173795	SLC25A52	4.10E-10	Clumping
30	rs113107469	18	29306737	SLC25A52	1.00E-19	
31	rs56069042	18	57914644	MC4R	1.20E-08	

Note: * To retain an independent set of SNPs for Mendelian randomization analysis, clumping threshold was set at linkage disequilibrium $r^2 < 0.01$, within 1Mb, the European panel, 1000 Genomes Project Phase 3.

Abbreviations: Chr, Chromosome; SNP, single nucleotide polymorphism.

Table S3 Association of instrumental SNPs with thyroid stimulating hormone and birth weight

SNP	Chr:Pos (GRCh37)	Nearest Gene	EA/OA	Association with maternal TSH			Association with offspring BW		
				Beta*	SE	*P*-value	Beta§	SE	*P*-value
rs10917469	1:19843576	CAPZB	A/G	0.111	0.009	4.0E-39	-0.002	0.006	0.78
rs334725	1:61610049	NFIA	A/G	0.174	0.015	2.4E-32	-0.005	0.010	0.65
rs17020122	1:108357391	VAV3	T/C	0.104	0.011	5.3E-20	0.001	0.007	0.89
rs13015993	2:217625523	IGFBP5	A/G	0.082	0.007	4.5E-32	-0.001	0.005	0.91
rs6724073	2:218236786	DIRC3	T/C	0.051	0.008	1.3E-10	0.005	0.005	0.28
rs1663070	3:12239852	SYN2	C/T	0.046	0.007	3.5E-11	-0.010	0.005	0.04
rs28502438	3:149220109	TM4SF4	T/C	0.034	0.006	3.7E-08	-0.004	0.004	0.42
rs13100823	3:185514088	IGF2BP2	C/T	0.041	0.007	6.8E-10	-0.010	0.005	0.04
rs59381142	3:193916181	HES1	G/A	0.058	0.008	1.7E-14	-0.003	0.005	0.59
rs11732089	4:149665602	NR3C2	T/C	0.115	0.008	1.7E-51	-0.003	0.005	0.53
rs2127387	5:76532571	PDE8B	A/G	0.144	0.006	1.1E-117	-0.004	0.004	0.40
rs1265091	6:31108129	PSORS1C1	T/C	0.057	0.009	3.2E-11	0.015	0.006	0.01
rs744103	6:43805362	VEGFA	A/T	0.092	0.007	6.7E-41	0.005	0.005	0.25
rs9497965	6:148521292	SASH1	T/C	0.044	0.006	9.8E-13	0.004	0.004	0.41
rs1079418	6:166047034	PDE10A	A/G	0.101	0.007	8.2E-53	-0.002	0.005	0.65
rs56009477	8:23356964	SLC25A37	A/G	0.052	0.008	3.7E-10	-0.001	0.006	0.93
rs2439301	8:32433013	NRG1	G/A	0.059	0.008	8.2E-15	-0.003	0.005	0.53
rs10957494	8:70365025	SULF1	G/A	0.040	0.007	1.1E-09	-0.002	0.005	0.72
rs118039499	8:133771635	TMEM71	A/C	0.184	0.024	2.0E-14	-0.019	0.014	0.17
rs10814915	9:4290544	GLIS3	T/C	0.042	0.006	5.1E-12	0.017	0.004	0.01
rs9298749	9:16214340	C9orf92	C/A	0.039	0.006	8.8E-10	0.004	0.005	0.39
rs11255790	10:8682180	GATA3	C/T	0.041	0.007	6.8E-10	0.002	0.005	0.66
rs4933466	10:89849519	PTEN	A/G	0.040	0.006	5.1E-10	-0.005	0.004	0.30

rs200574439	10:101283330	NKX2-3	C/A	0.047	0.006	3.7E-13	0.004	0.004	0.40
rs12284404	11:45228686	PRDM11	G/A	0.067	0.007	2.5E-22	-0.004	0.005	0.43
rs4445669	11:115045237	CADM1	C/T	0.040	0.006	5.8E-11	-0.003	0.004	0.54
rs7329958	13:24782080	SPATA13	C/T	0.044	0.007	1.1E-11	0.001	0.005	0.88
rs398745	14:36536181	MBIP	C/A	0.052	0.006	4.0E-17	0.003	0.004	0.49
rs12893151	14:81619945	TSHR	C/A	0.062	0.008	1.0E-15	-0.002	0.005	0.77
rs8015085	14:93585331	ITPK1	A/G	0.067	0.008	2.4E-18	0.008	0.006	0.15
rs17477923	15:49711185	FAM227B	T/C	0.083	0.007	2.6E-33	-0.008	0.005	0.10
rs13329353	15:89113877	DET1	T/C	0.061	0.007	5.2E-21	0.002	0.005	0.62
rs1045476	16:4015313	ADCY9	A/G	0.049	0.008	2.4E-09	0.005	0.006	0.35
rs30227	16:14405428	MIR365A	C/T	0.047	0.006	7.6E-14	-0.007	0.004	0.12
rs17767491	16:79745487	MAF	A/G	0.088	0.007	3.4E-42	0.006	0.005	0.22
rs77819282	17:44762589	NSF	A/G	0.045	0.007	1.1E-09	-0.005	0.005	0.36
rs1157994	17:59338574	BCAS3	G/A	0.090	0.016	5.3E-09	0.017	0.011	0.12
rs1042673	17:70121339	SOX9	G/A	0.055	0.006	3.6E-19	-0.007	0.004	0.10
rs4804413	19:7222655	INSR	T/C	0.053	0.006	8.6E-18	0.013	0.004	0.01
rs1203944	20:22596879	FOXA2	C/T	0.051	0.007	2.4E-12	0.011	0.005	0.03

Note: * Beta represented effect size in the unit of standard deviation of TSH concentrations;

§ Beta represented the maternal effect on offspring birth weight adjusted for the correlation between fetal and maternal genotypes, in the unit of Z-score of birth weight. (Warrington NM, et al. *Nat Genet*, 2019, 51:804-14. doi: 10.1038/s41588-019-0403-1)

Abbreviations: BW, birth weight; Chr:Pos, Chromosome and position; EA/OA, effect allele/reference allele; SE, standard error for Beta; SNP, single nucleotide polymorphism; TSH, thyroid stimulating hormone.

Table S4 Association of instrumental SNPs with free thyroxine and birth weight

SNP	Chr:Pos (GRCh37)	Nearest Gene	EA/OA	Association with maternal FT4			Association with offspring BW		
				**Beta***	**SE**	***P*-value**	**Beta§**	**SE**	***P*-value**
rs2235544	1:54375570	DIO1	A/C	0.139	0.007	4.20E-101	0.001	0.004	0.88
rs4954192	2:135632981	ACMSD	C/T	0.041	0.007	8.40E-09	-0.004	0.004	0.38
rs6785807	3:181718601	SOX2-OT	G/A	0.059	0.009	2.50E-10	-0.008	0.006	0.18
rs6854291	4:170992760	AADAT	A/G	0.117	0.011	1.30E-24	0.004	0.007	0.57
rs10946313	6:19381386	ID4	T/C	0.046	0.007	2.30E-11	-0.007	0.004	0.12
rs9356988	6:25777481	SLC17A4	G/A	0.051	0.007	3.60E-12	0.008	0.005	0.10
rs17185536	6:100620931	LOC728012	T/C	0.073	0.008	1.90E-19	-0.002	0.005	0.65
rs67583169	8:61212179	CA8	C/G	0.061	0.01	1.00E-10	0.009	0.006	0.15
rs10119187	9:4223660	GLIS3	T/C	0.05	0.009	4.10E-09	-0.001	0.006	0.90
rs10739496	9:100552559	FOXE1	T/C	0.078	0.007	4.20E-30	0.006	0.005	0.20
rs10818937	9:127015440	NEK6	C/T	0.048	0.007	1.30E-11	-0.003	0.005	0.54
rs4842131	9:139092679	LHX3	C/T	0.104	0.008	7.70E-44	-0.001	0.004	0.74
rs11039355	11:47725306	FNBP4	C/T	0.039	0.007	3.50E-08	0.003	0.005	0.56
rs4149056	12:21331549	SLCO1B1	C/T	0.051	0.009	1.30E-08	0.010	0.006	0.09
rs225014	14:80669580	DIO2	T/C	0.054	0.007	1.80E-15	0.004	0.004	0.39
rs11626434	14:101998443	DIO3OS	C/G	0.058	0.007	4.10E-17	-0.006	0.005	0.23

rs12907106	15:63873658	USP3	G/C	0.041	0.007	3.70E-08	-0.006	0.005	0.26
rs8063103	16:12703395	SNX29	G/C	0.052	0.009	1.60E-08	0.002	0.006	0.75
rs11078333	17:16049626	NCOR1	A/T	0.051	0.007	9.90E-13	-0.004	0.004	0.32
rs113107469	18:29306737	SLC25A52	T/C	0.2	0.022	1.00E-19	0.000	0.012	0.99
rs56069042	18:57914644	MC4R	A/G	0.106	0.019	1.20E-08	-0.012	0.012	0.32

Note: * Beta represented effect size in the unit of standard deviation of FT4 concentrations;

† Beta represented the maternal effect on offspring birth weight adjusted for the correlation between fetal and maternal genotypes, in the unit of Z-score of birth weight. (Warrington NM, et al. *Nat Genet*, 2019, 51:804-14. doi: 10.1038/s41588-019-0403-1)

§ For rs11039355, the proxied SNP was used (rs11604825, $r^2 = 0.96$, D’ = 1.0) in the association with birth weight.

Abbreviations: BW, birth weight; Chr:Pos, Chromosome and position; EA/OA, effect allele/reference allele; FT4, free thyroxine; SE, standard error for Beta; SNP, single nucleotide polymorphism.
